# Supplementary material for: Cross-talks of glycosylphosphatidylinositol biosynthesis with glycosphingolipid biosynthesis and ER-associated degradation
Source: Nat Commun. 2020 Feb 13;11:860. doi: 10.1038/s41467-020-14678-2 (PMC7018848; doi:10.1038/s41467-020-14678-2)
Supplement: Supplementary file 1 — Supplementary Information [file 41467_2020_14678_MOESM1_ESM.pdf]

Supplementary Information

Cross-talks of glycosylphosphatidylinositol biosynthesis with glycosphingolipid biosynthesis and ER-associated degradation

Wang et al.

Supplementary Figures

Supplementary Figure 1

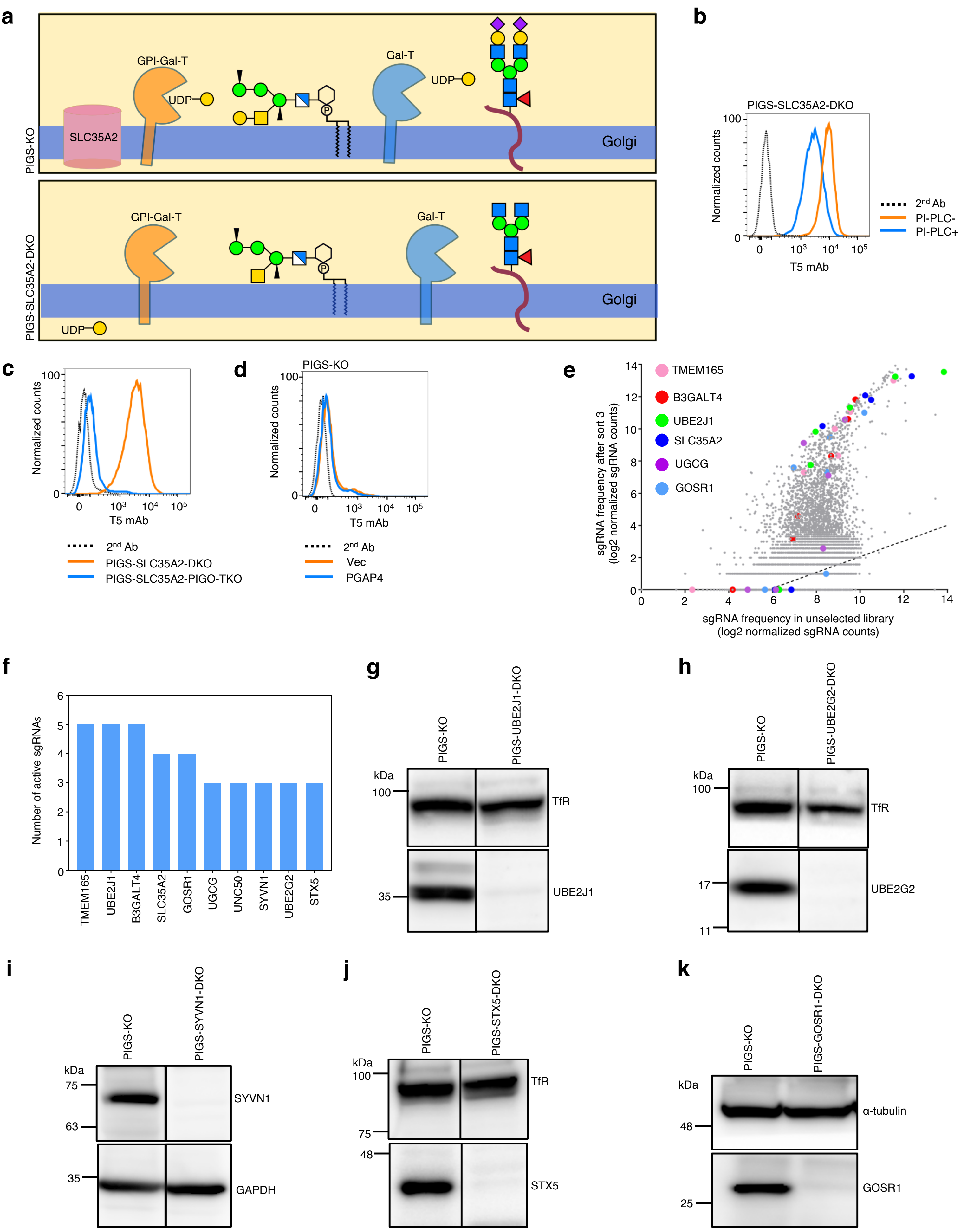

Supplementary Figure 1 CRISPR-Based genetic screen identified genes involved in galactosylation of GPI. Related to Fig. 1 and 2. **a** Expected galactosylation profiles of free GPI-GalNAc and *N*-glycan in PIGS-KO and PIGS-SLC35A2-DKO HEK293 cells. Related to Fig. 1b. **b** Flow cytometry analysis of PIGS-SLC35A2-DKO cells treated with or without PI-PLC. Cells were stained with T5 mAb plus Alexa Fluor 488 (AF488)-secondary antibody. **c** Flow cytometry analysis of PIGS-SLC35A2-DKO and PIGS-SLC35A2-PIGO-TKO cells. Cells were stained by T5 mAb plus AF647-secondary antibody. **d** Flow cytometry analysis of PIGS-KO cells transiently expressing PGAP4. Cells were stained by T5 mAb plus AF647-secondary antibody. **e** Individual sgRNAs targeting the candidate genes were highly enriched in the sort3 cells as compared with the unsorted cells. The dashed line represents the linear regression line of best fit. Related to Figure 1e. **f** Active sgRNA number of top identified genes. Related to Fig. 1e and Supplementary Fig. 1e. **g-k** Confirmation of UBE2J1-KO, UBE2G2-KO, SYVN1-KO, STX5-KO, and GOSR1-KO by Western blotting. TfR, GAPDH, and α-tubulin: loading controls.

Supplementary Figure 2

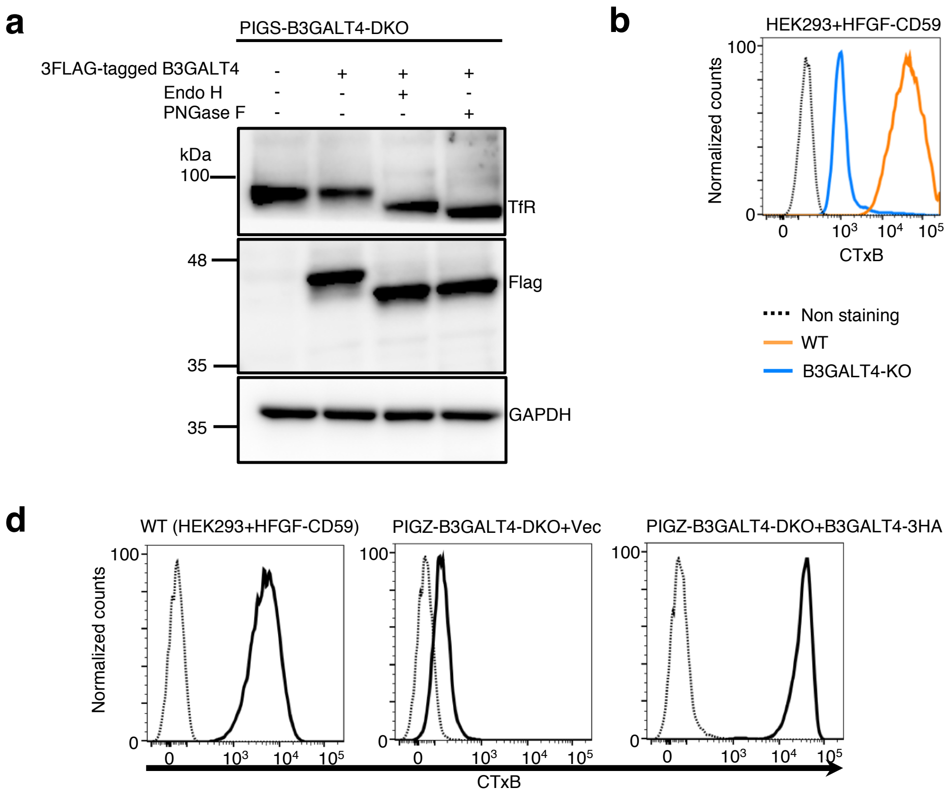

Supplementary Figure 2 B3GALT4 transfers Gal to both GM2 and GPI-GalNAc. Related to Fig. 3. **a** Western blotting of 3FLAG tagged human B3GALT4 stably expressed in PIGS-B3GALT4-DKO cells. Lysates were treated with or without Endo H or PNGase F before Western blotting. GAPDH, a loading control. **b** Confirmation of B3GALT4-KO in HEK293 cells stably expressing HFGF-CD59. Cells stained with AF488-CTxB were analyzed by flow cytometry. **c** Confirmation of stable expression of 3HA tagged human B3GALT4 in B3GALT4-PIGZ-DKO HEK293 cells that are stably expressing HFGF-CD59. Cells transfected with vector or 3HA tagged B3GALT4 were analyzed by western blotting against anti-HA antibody. TfR, a loading control. **d** CTxB staining of B3GALT4-PIGZ-DKO HEK293 cells stably expressing Vec or 3HA tagged B3GALT4. Cells stained with AF488-CTxB were analyzed by flow cytometry.

Supplementary Figure 3

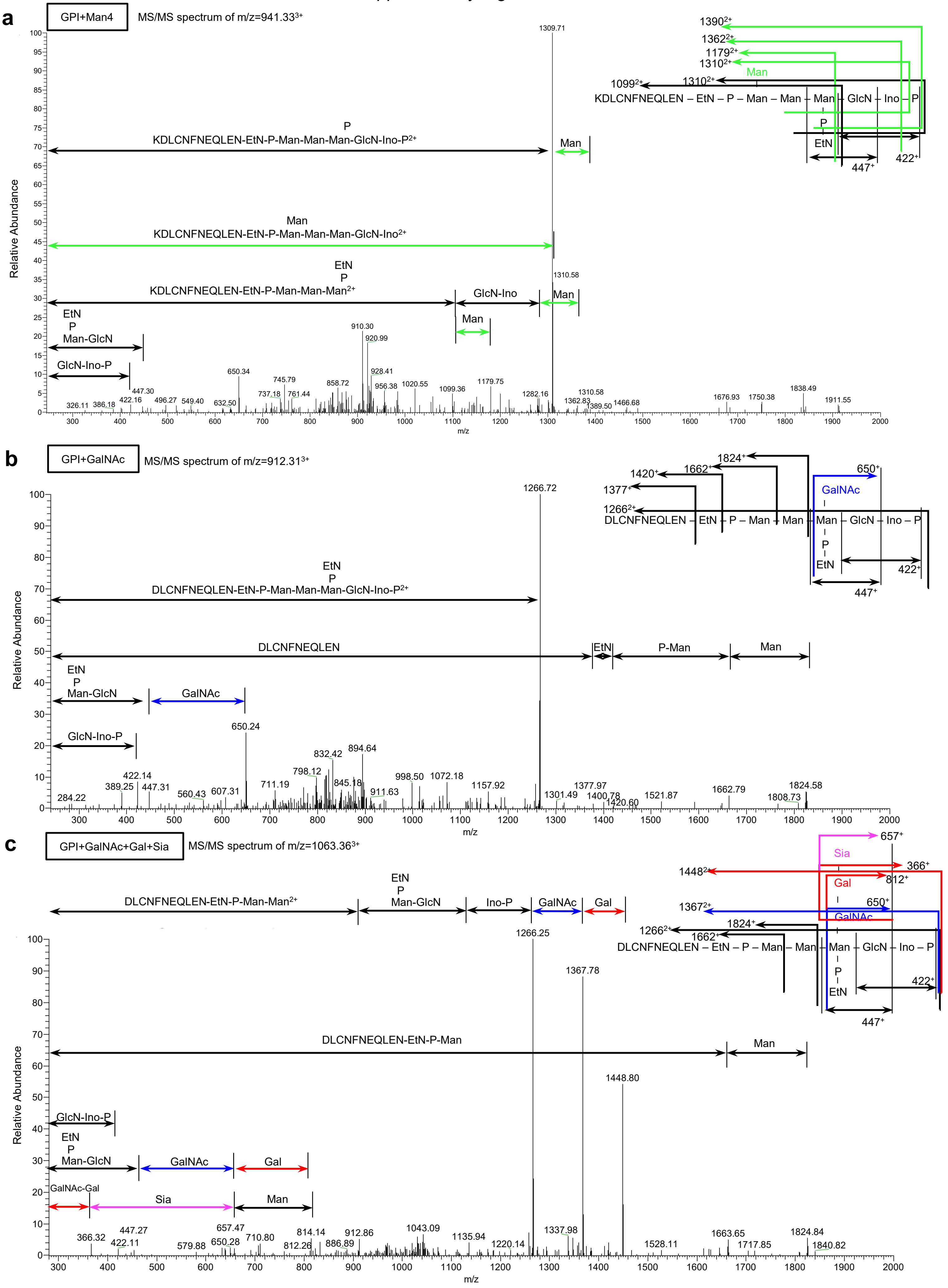

Supplementary Figure 3

d GPI+GalNAc+Hex — MS/MS spectrum of m/z=966³⁺

GPI+GalNAc-Gal

m/z=1347²⁺

Gal
|
GalNAc
|
DLCNFNEQLEN-EtN-P-Man-Man-Man-GlcN-Ino²⁺

GPI+GalNAc+Man4

m/z=1347²⁺

Man GalNAc
| |
DLCNFNEQLEN-EtN-P-Man-Man-Man-GlcN-Ino²⁺

Man
|
DLCNFNEQLEN-EtN-P-Man-Man-Man-GlcN-Ino-P²⁺
|
P
|
EtN

e GPI+GalNAc+Man4 — MS/MS spectrum of m/z=966.00³⁺

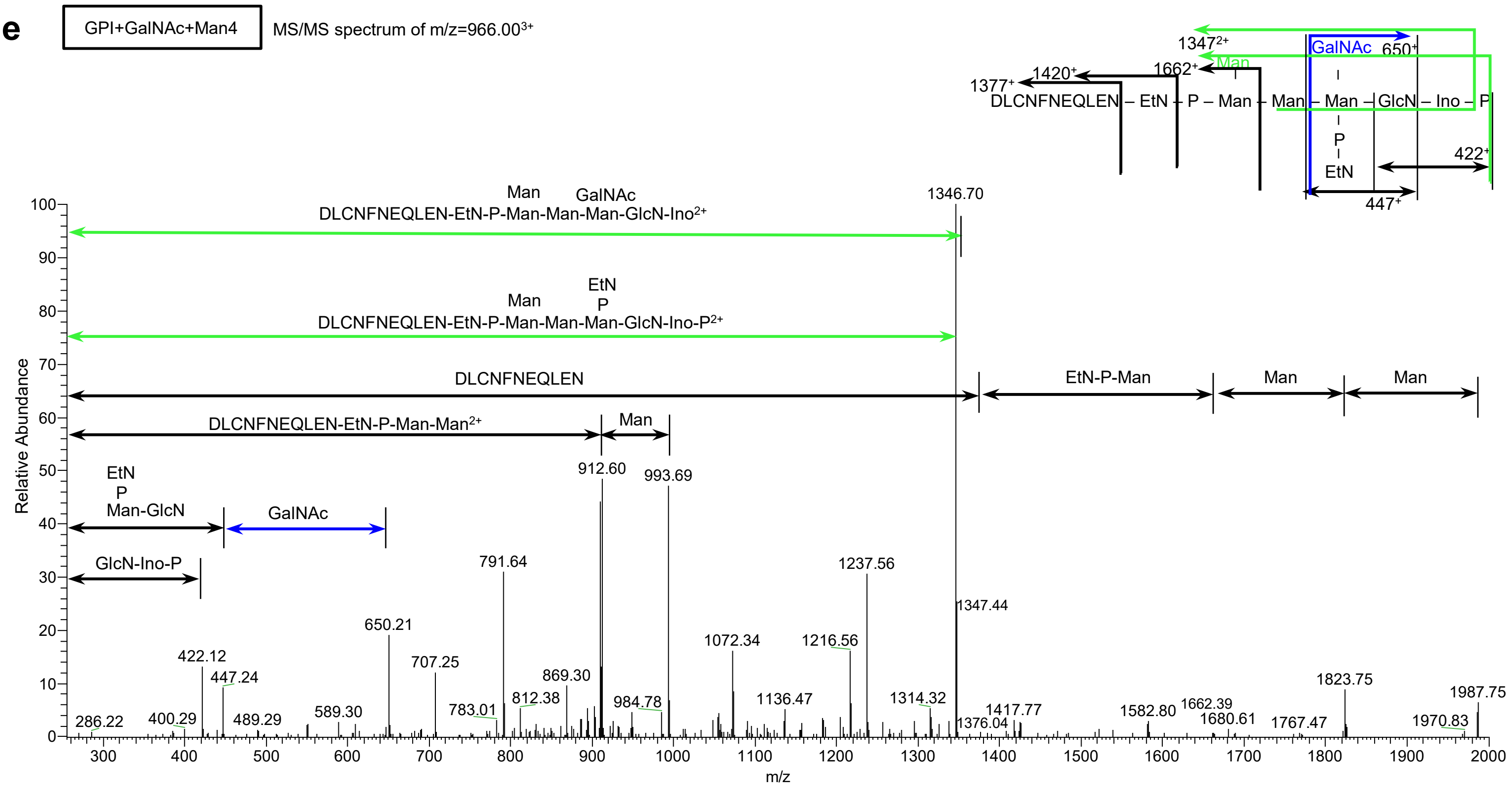

f GPI+GalNAc+Gal — MS/MS spectrum of m/z=966.33³⁺

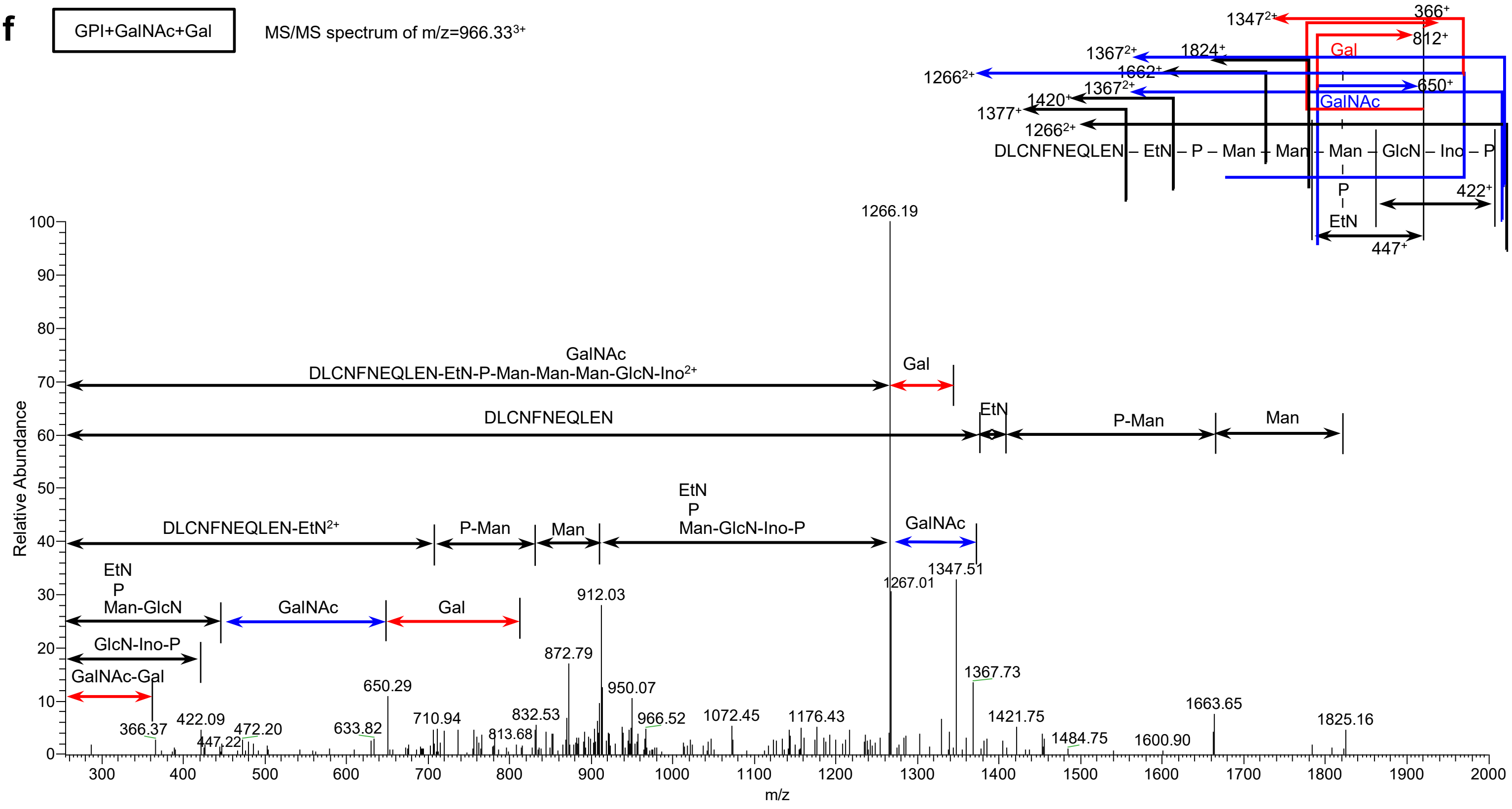

Supplementary Figure 3

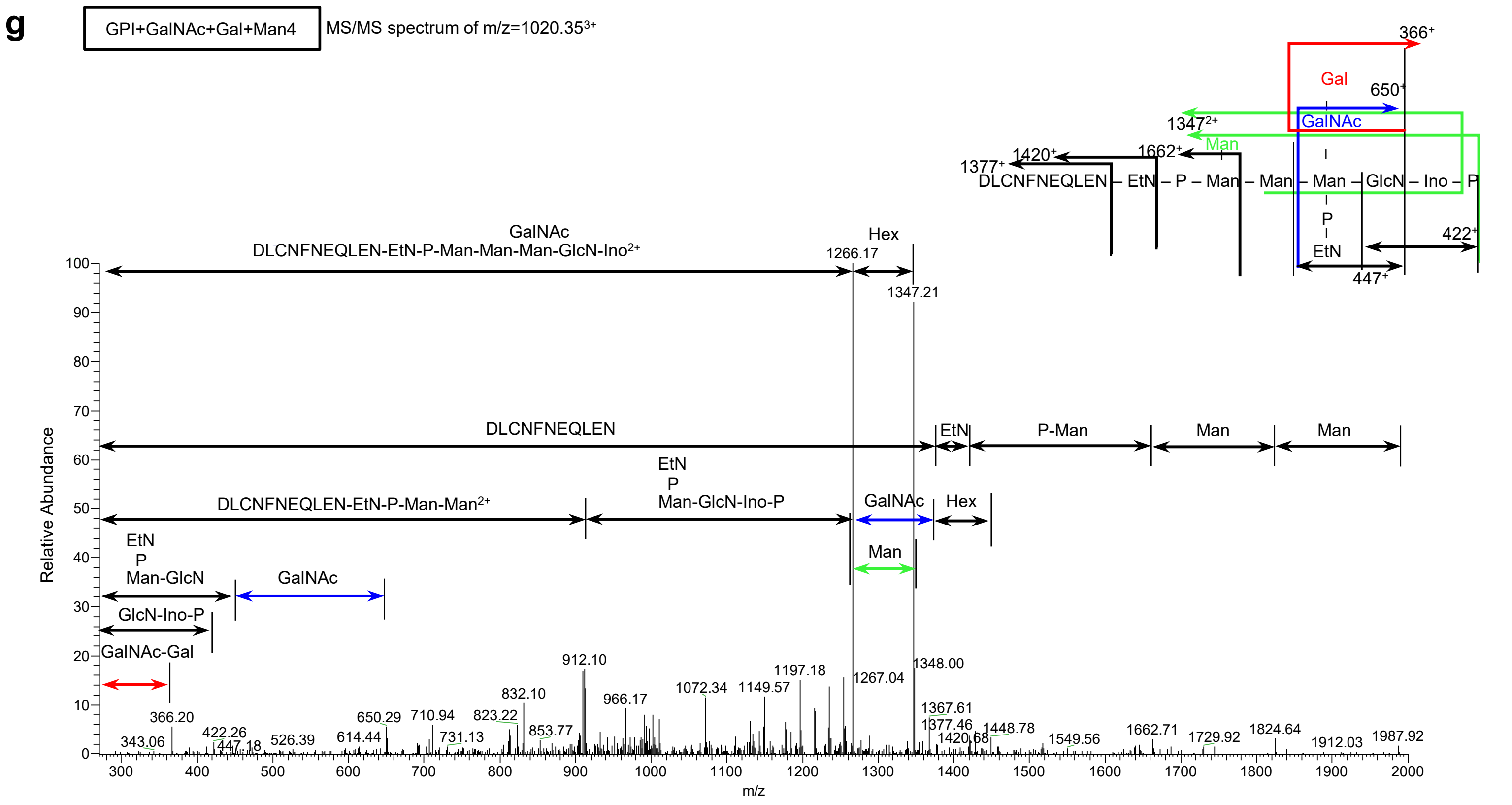

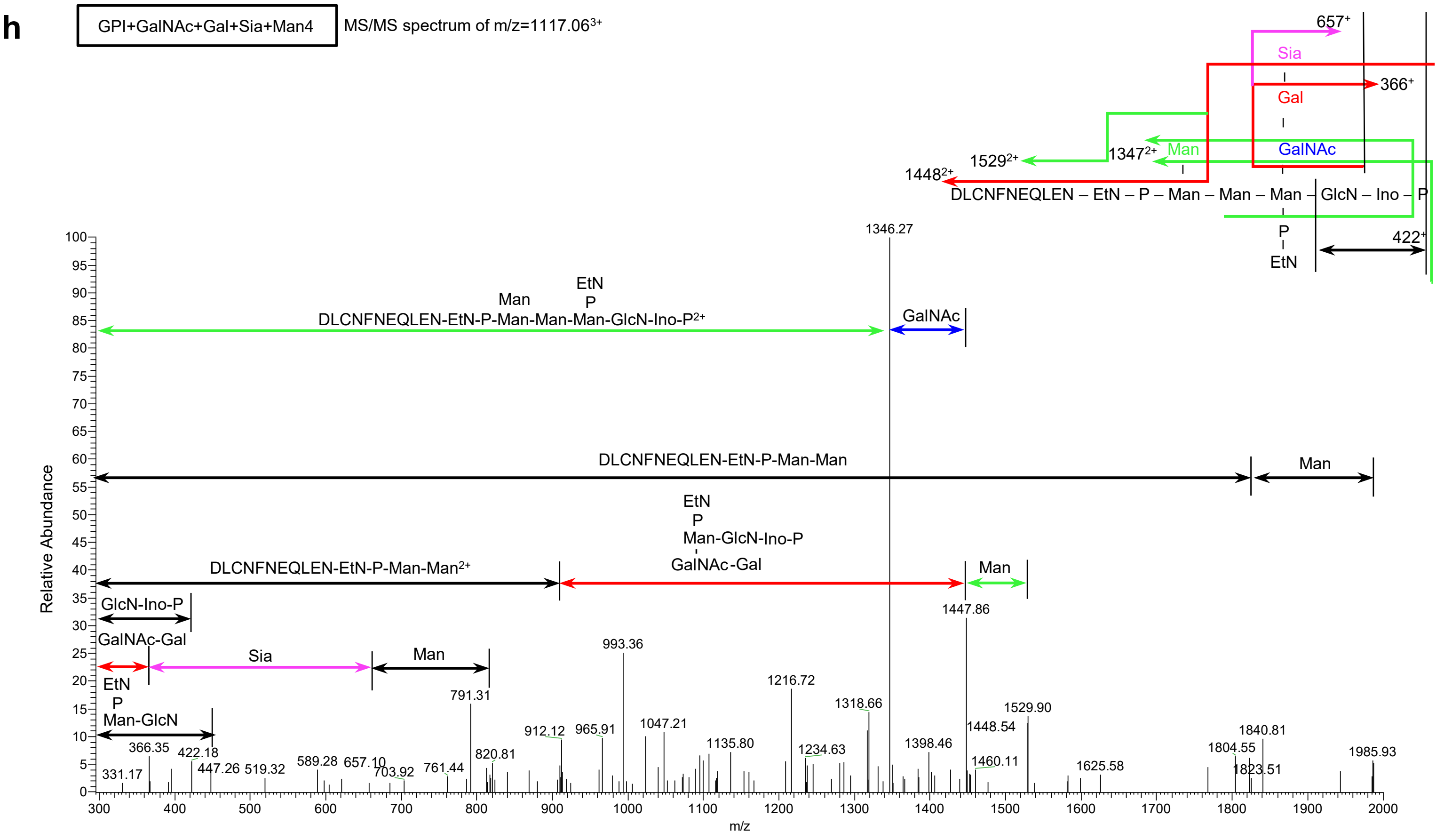

Supplementary Figure 3 ESI-MS/MS analysis of GPI in tagged CD59 derived from HEK293 Cells. Related to Fig. 3e and f, and Supplementary Table 2. **a** 941.33^{3+} for GPI core + Man4 with an additional lysine residue from HEK293 cells (WT). **b** 912.31^{3+} for GPI core + GalNAc from WT cells. **c** 1063.36^{3+} for GPI core + GalNAc + Gal + Sia from WT cells. **d** Fragment ions of m/z 1347^{2+} from MS/MS spectra of 966^{3+} for GPI core + GalNAc + Hex. One peak of m/z 1347^{2+} indicates GPI core + GalNAc + Gal, while two peaks of m/z 1347^{2+} suggest GPI core + GalNAc + Man4. **e** 966.00^{3+} for GPI core + GalNAc + Gal from WT cells. **f** 966.33^{3+} for GPI core + GalNAc + Man4 from B3GALT4-PIGZ-DKO cells expressing an empty vector. **g** 1020.35^{3+} for GPI core+ GalNAc + Gal + Man4 from WT cells. **h** 1117.06^{3+} for GPI core+ GalNAc + Gal + Sia + Man4 from B3GALT4-PIGZ-DKO cells expressing 3HA tagged B3GALT4. For **a**, **b**, **c**, **e**, **f**, **g**, and **h**, MS/MS spectra of C-terminal tryptic peptides with GPI derived from HFGF-CD59 released from cells by PI-PLC-treatment are displayed.

Supplementary Figure 4

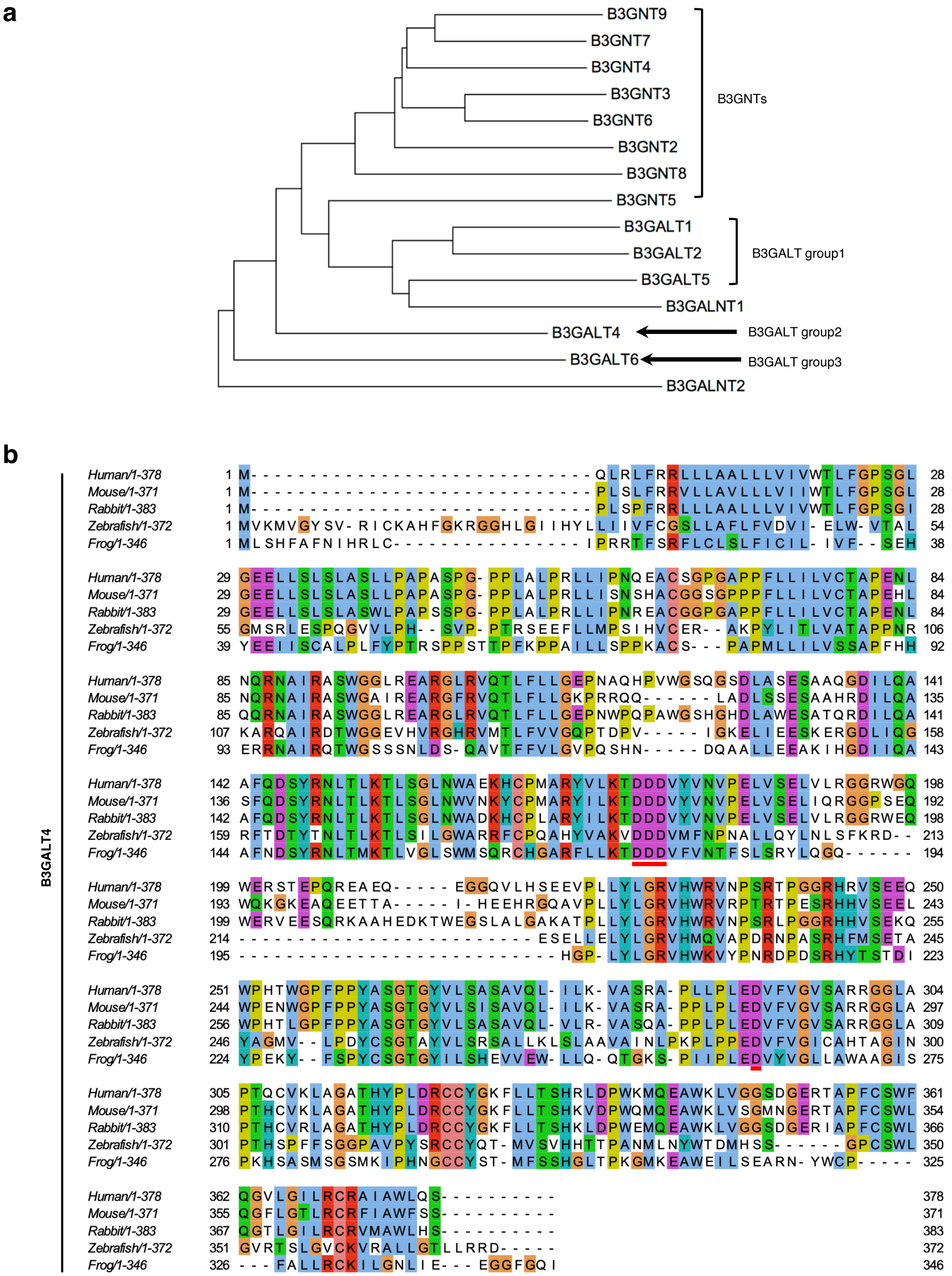

Supplementary Figure 4

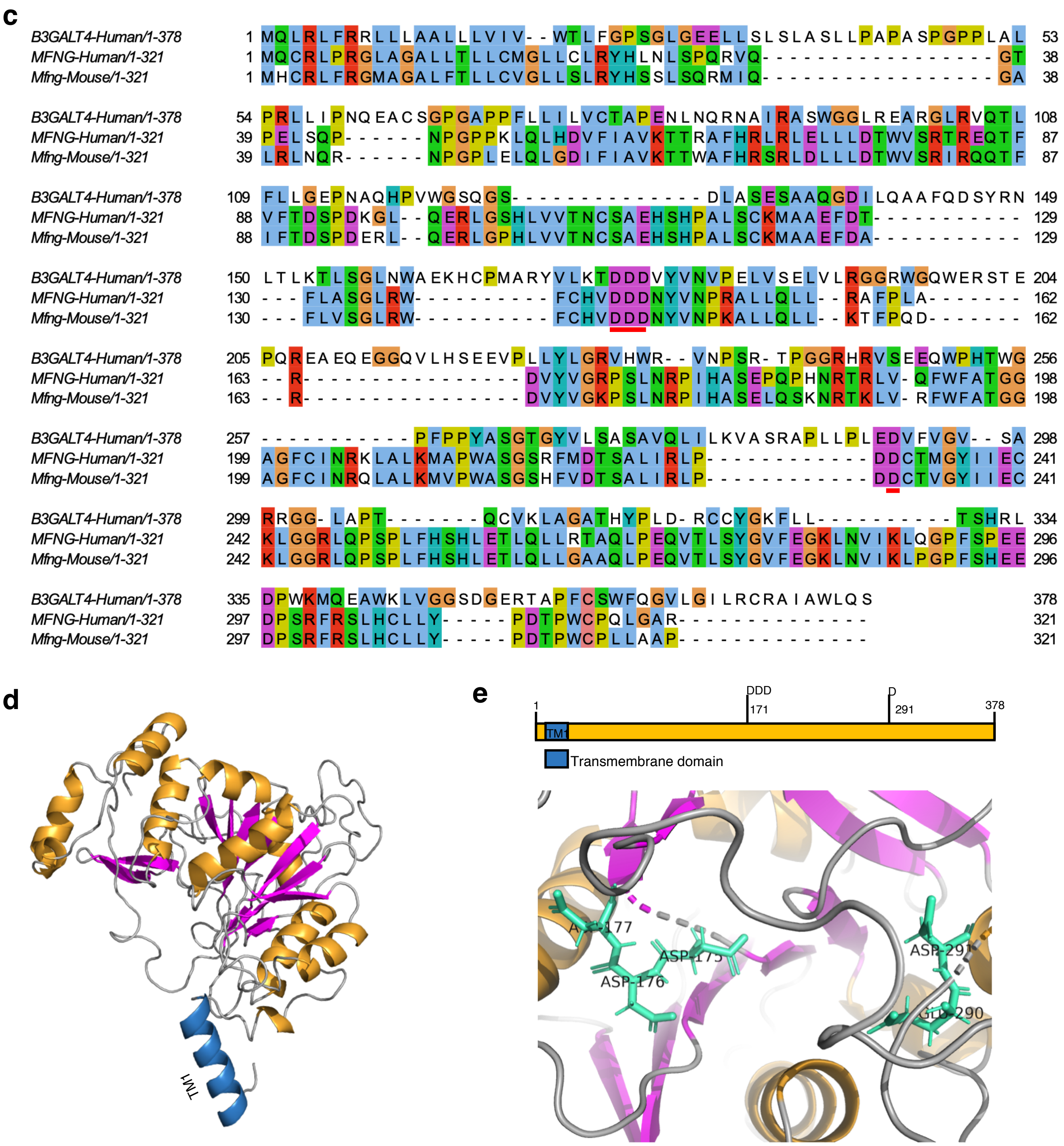

Supplementary Figure 4 Characterization of the catalytic region of B3GALT4 for GM1 synthesis and GPI side-chain galactosylation. Related to Fig. 3g and h. **a** Phylogenetic tree of human beta 3-glycosyltransferases shows that beta 3-galactosyltransferases are classified into three B3GALT groups. The sequences of human beta 3-glycosyltransferases are from Uniprot; B3GNT2 (Q9NY97); B3GNT3 (Q9Y2A9); B3GNT4 (Q9C0J1); B3GNT5 (Q9BYG0); B3GNT6 (Q6ZMB0); B3GNT7 (Q8NFL0); B3GNT8 (Q7Z7M8); B3GNT9 (Q6UX72); B3GALT1 (Q9Y5Z6); B3GALT2 (O43825); B3GALT4 (O96024); B3GALT5 (Q9Y2C3); B3GALT6 (Q96L58); B3GALNT1 (O75752); B3GALNT2 (Q8NCR0). The phylogenetic tree was generated by neighbor-joining using MEGA7[1]. **b** B3GALT4 primary sequence homology. The T-coffee program was used to generate a sequence alignment of B3GALT4 and colored by the Clustal style in the Jalview[2]. B3GALT4 sequences are from NCBI; human (NP_003773.1); mouse (NP_062293.1); rabbit (XP_002714663.1); zebrafish (XP_005167417.1); frog (XP_002938731.1). The DDD motif (D175-D176-D177 in human sequence) and D291 (in human sequence) are underlined by solid red lines. **c** Sequence alignment of human B3GALT4 with human and mouse MFNG (Beta-1,3-N-acetylglucosaminyltransferase manic fringe). The DDD motif (D175-D176-D177 in B3GALT4 sequence) and D291 (B3GALT4 sequence) are underlined by solid red lines. Sequences are from Uniprot; human B3GALT4 (O96024); mouse MFNG (O09008); human MFNG (O00587). **d** Three-dimensional structural model of B3GALT4 predicted by I-TASSER. The first transmembrane domain (TM1) is shown in blue, the β-sheets are shown in magenta, and the α-helices are shown in yellow. **e** Enlarged view of catalytic regions of B3GAL4 model. D175, D176, D177 and D291 are shown with sticks.

Supplementary Figure 5

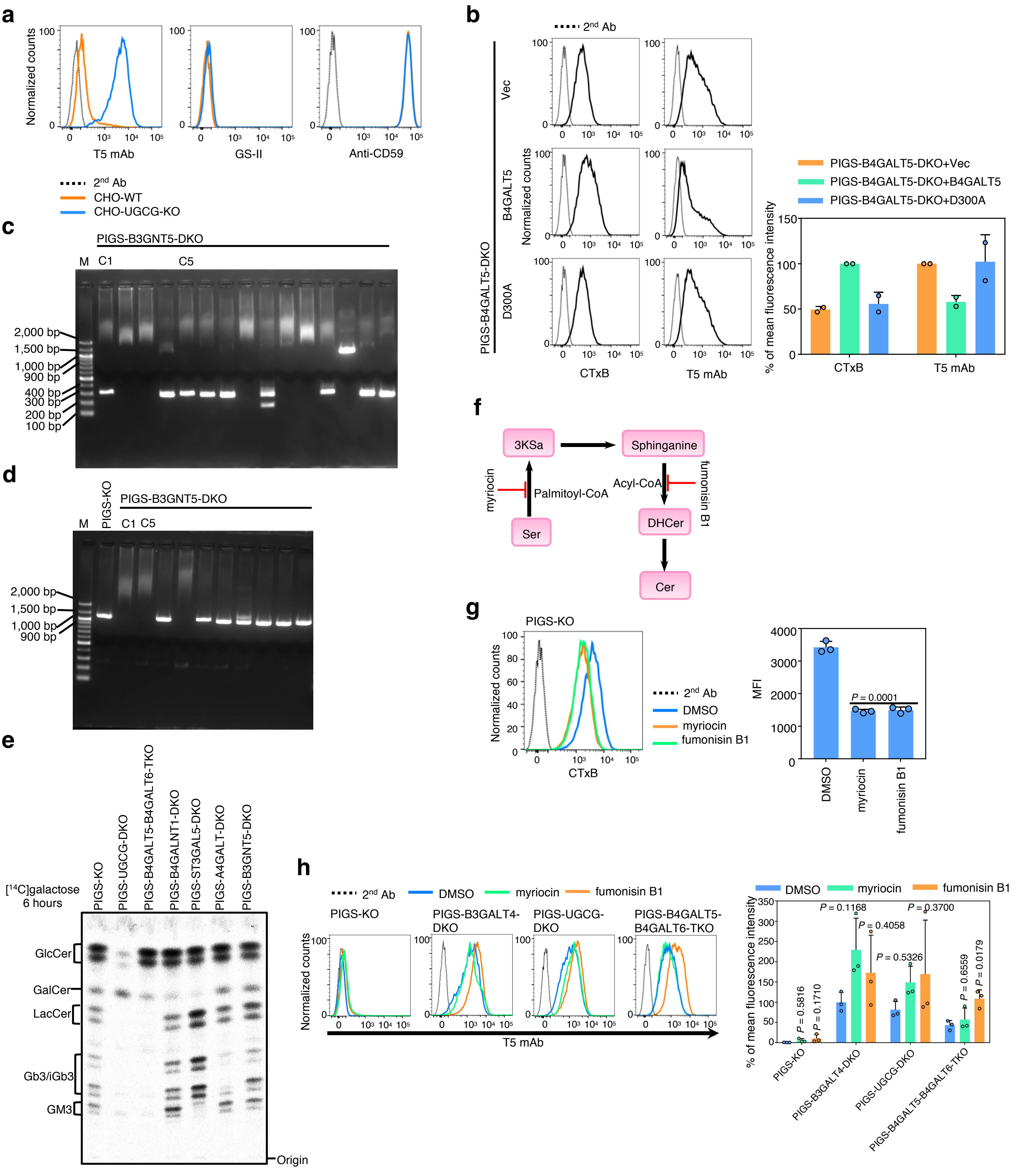

Supplementary Figure 5 Biosynthesis of lactosylceramides is required for Gal modification of GPI-GalNAc. Related to Fig. 4. **a** Flow cytometry analysis of CHO cells and CHO-UGCG-KO cells. Cells were stained with T5 mAb plus AF647-secondary antibody, biotin conjugated anti-CD59 mAb plus PE-Streptavidin, or AF647-GS-II lectin. **b** Left. Flow cytometry analysis of PIGS-B4GALT5-DKO HEK293 cells transiently expressing pME-B4GALT5-3HA (B4GALT5) or pME-B4GALT5-D300A-3HA (D300A). Cells were stained with AF488-CTxB or T5 mAb plus AF647-secondary antibody. Right. Quantitative data of MFI from two independent experiments (mean ± SD, n=2). **c** and **d** KO of B3GNT5 was confirmed by PCR using two primer sets. The expected amplicon for B3GNT5 is 1208 bp for PIGS-KO and 309 bp for PIGS-B3GNT5-DKO in **c**. The expected amplicon for B3GNT5 is 1137 bp for PIGS-KO and no amplification for PIGS-B3GNT5-DKO in **d**. PIGS-B3GNT5-DKO-C5 was further analyzed in Figure 4g. **e** High-performance thin-layer chromatography profile of [^{14}C]galactose-labelled GSLs from HEK293 cells. Related to Figure 4h. **f** Ceramide (Cer) biosynthesis and inhibitors which block Cer generation. **g** Left: PIGS-KO HEK293 cells treated with myriocin or fumonisin B1 were stained with CTxB. Right: Quantitative data of MFI from three independent experiments (mean ± SD, n=3). *P* values are from one-way ANOVA followed by Dunnett's test for multiple comparisons to no treatment control (DMSO). Related to Figure 4c and d. **h** Left: PIGS-KO, PIGS-B3GALT4-DKO, PIGS-UGCG-DKO, and PIGS-B4GALT5-B4GALT6-TKO cells treated with myriocin or fumonisin B1 for 3 days were stained with T5 mAb. Right: Quantitative data of MFI from three independent experiments (mean ± SD, n=3); *P* values are from one-way ANOVA followed by Dunnett's test for multiple comparisons to no treatment control (DMSO). Related to Figure 4c and d. Source data for **b**, **g** and **h** are provided as a Source Data file.

Supplementary Figure 6

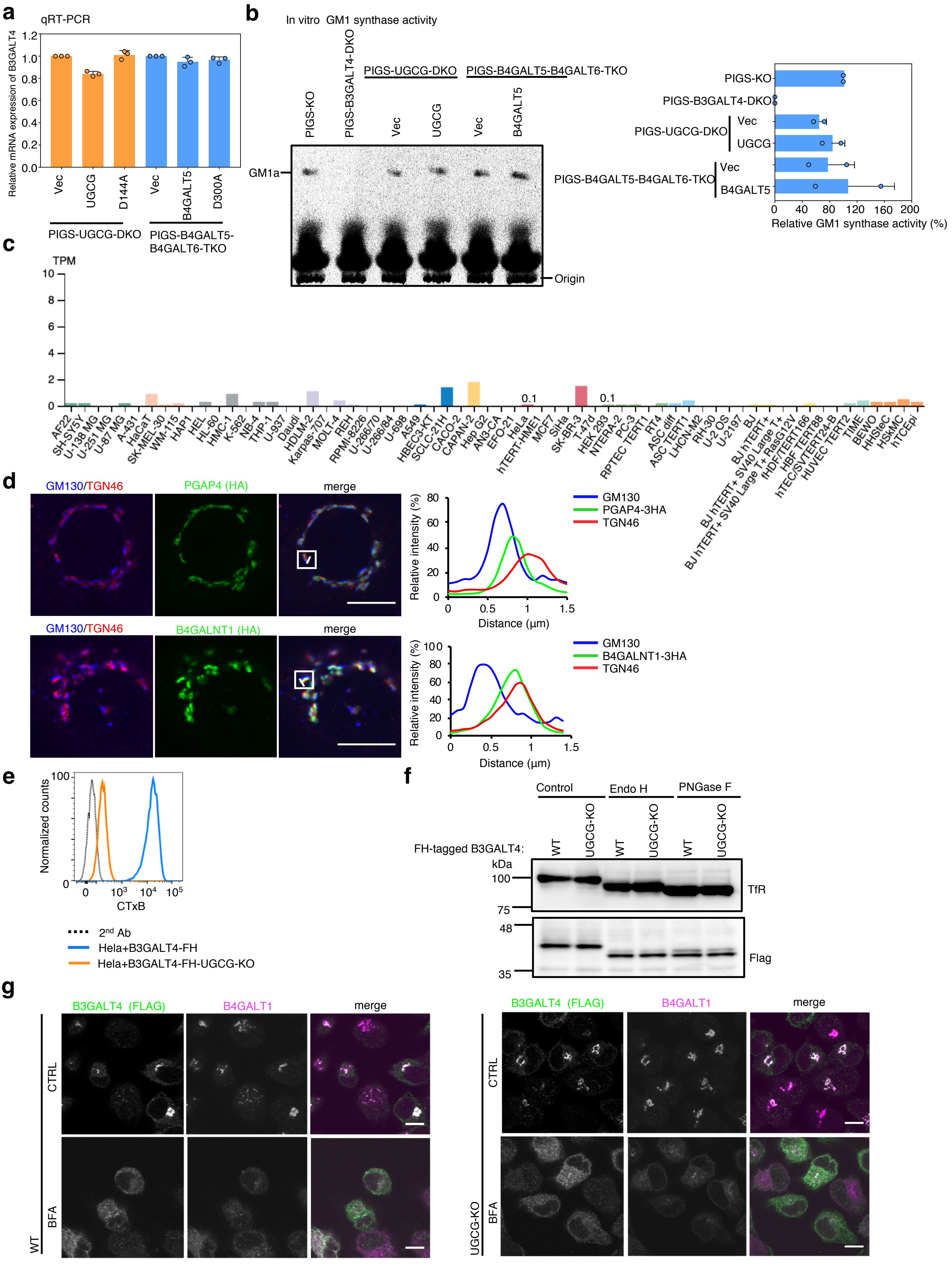

Supplementary Figure 6 Lack of LacCer does not affect expression and intra-Golgi localization of B3GALT4. Related to Fig. 5 and 6. **a** Relative expression of B3GALT4 mRNA from PIGS-UGCG-DKO cells stably expressing WT or D144A mutant of UGCG (orange), and PIGS-B4GALT5-B4GALT6-TKO cells stably expressing WT or D300A mutant of B4GALT5 (blue). Quantitative data from three independent analyses by qRT-PCR (mean ± SD, n=3). **b** Measurement of GM1 synthase activity in vitro. Left: Cell lysates were incubated with GM2 and UDP-[6-^{3}H] Galactose for 2 hours, and labelled products were separated by HPTLC. Strong spots below GM1 are UDP-galactose. Right: Quantitative data from two reactions (mean ± SD, n=2). **c** Transcript abundance of B3GALT4 in various cell lines, taken from the Human Protein Atlas (HPA)[3]. The RNA-seq results in the HPA are reported as number of Transcripts per Kilobase Million (TPM), a TPM value of 1.0 is defined as a threshold for expression of the corresponding protein. The TPM values for B3GALT4 in HeLa and HEK293 cells, 0.1 and 0.1, are indicated. **d** Linescan (right) was obtained from a HeLa cell fixed and stained as described to detecting the HA tag (green) for PGAP4 (upper) or B4GALNT1 (lower) and a cis-Golgi marker (GM130; blue) and a TGN marker (TGN46; red). Linescan shows fluorescence intensity along a portion of the white line overlaying the image (left). Scale bar, 10 μm. Related to Figure 5b. **e** Confirmation of UGCG-deficiency in HeLa cells stably expressing FH tagged B3GALT4, WT or UGCG-KO cells were stained with AF488-CTxB. **f** Western blotting of B3GALT4 in WT or UGCG-KO HeLa cells. Lysates were treated by Endo H or PNGase F and analyzed by Western blotting. TfR, a loading control. **g** WT (left) and UGCG-KO (right) HeLa cells stably expressing FH tagged B3GALT4 were treated with or without Brefeldin A (BFA), fixed and stained for FLAG tag, as well as B4GALT1 (trans-Golgi marker). Scale bar, 10 μm. Source data for **a** and **b** are provided as a Source Data file.

Supplementary Figure 7

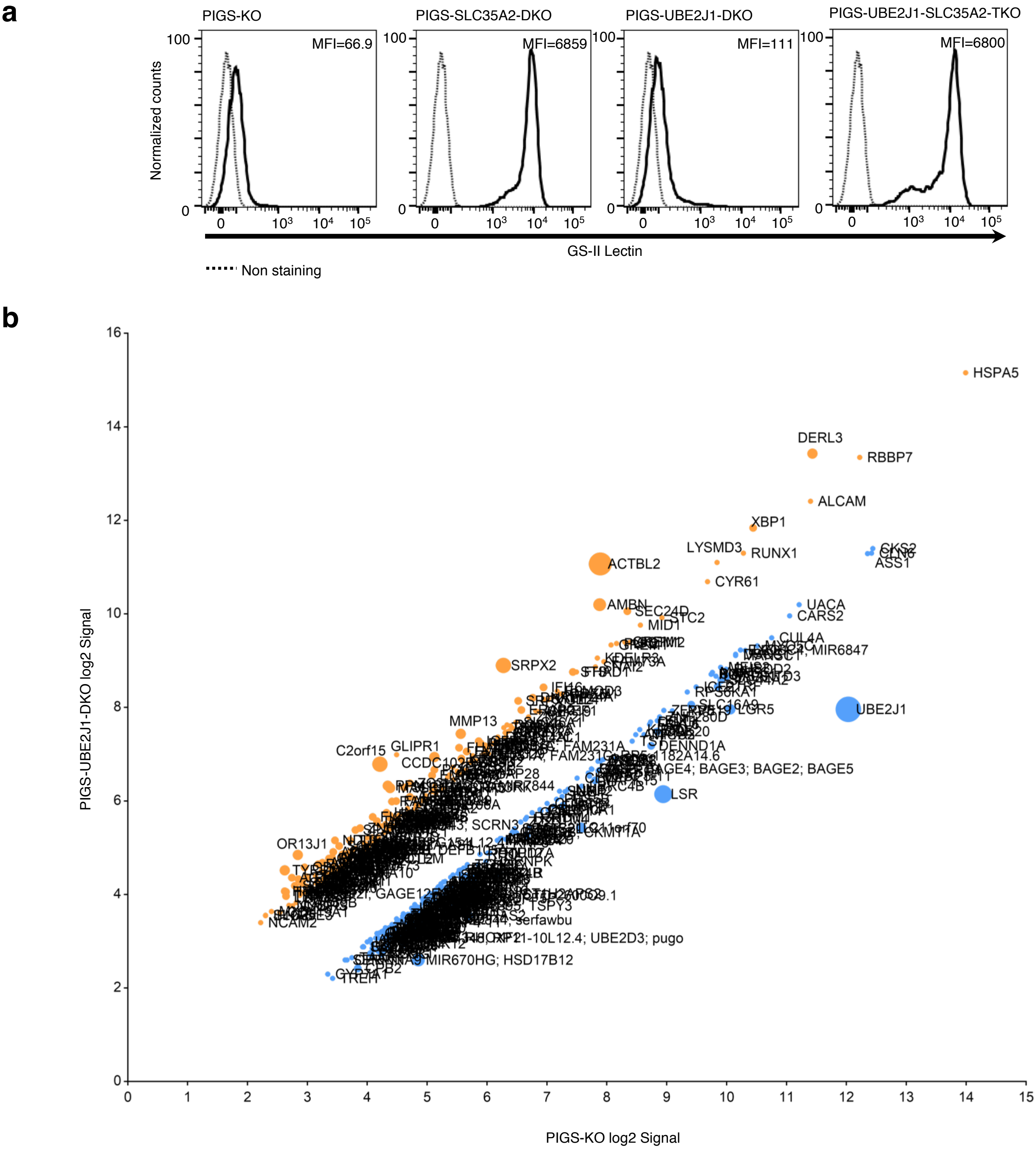

Supplementary Figure 7 Biosynthesis of GPI was under regulation by ERAD in PIGS-KO cells. **a** Confirmation of SLC35A2-deficiency in HEK293-PIGS-UBE2J1-SLC35A2-TKO cells. Cells were probed for *N*-glycan profile using GS-II lectin staining and analyzed by flow cytometry. MFI values are indicated. Related to Fig. 7b. **b** Microarray of HEK293-PIGS-KO and HEK293-PIGS-UBE2J1-DKO cells, genes whose expression fold change higher than 2 or lower than -2 are shown. Related to Fig. 7h and Supplementary Data 4.

Supplementary Figure 8

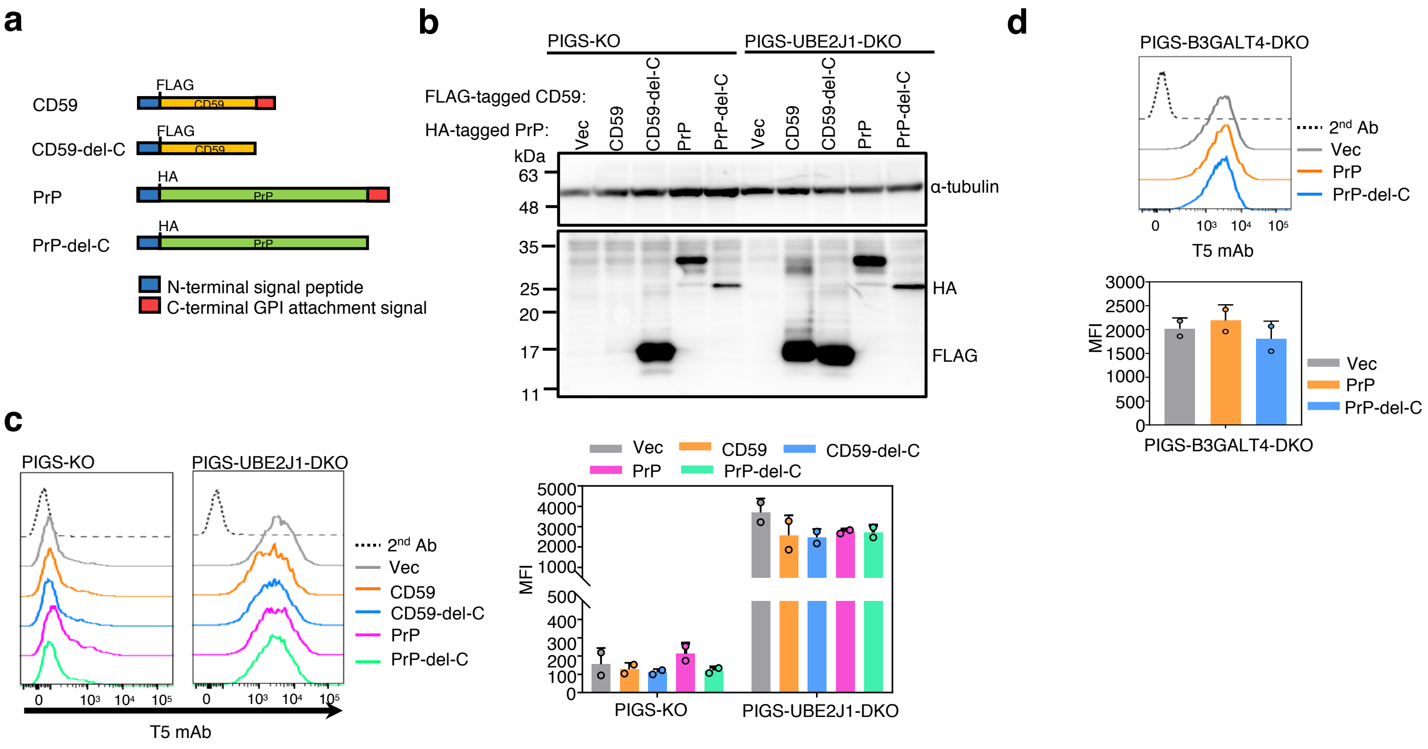

Supplementary Figure 8 Transfection of CD59 and prion did not upregulate GPI biosynthesis. a Schematic of FLAG tagged human CD59 and HA tagged Prion (PrP) protein with or without C-terminal GPI attachment signal peptide. **b** Western blotting of FLAG-tagged CD59 or CD59 without C-terminal GPI anchor attachment signal peptide (CD59-del-C), and HA-tagged prion protein (PrP) or PrP-del-C in PIGS-KO and PIGS-UBE2J1-DKO HEK293 cells. Lysates were analyzed by Western blotting. α-tubulin, a loading control. **c** Left: PIGS-KO or PIGS-UBE2J1-DKO cells expressing Vec, CD59, CD59-del-C, PrP, or PrP-del-C were stained with T5 mAb. Right: Quantitative data of MFI from two independent experiments (mean ± SD, n=2). **d** Top: PIGS-B3GALT4-DKO cells expressing Vec, PrP, or PrP-del-C were stained with T5 mAb. Bottom: Quantitative data of MFI from two independent experiments (mean ± SD, n=2). Source data for **c** and **d** are provided as a Source Data file.

Supplementary Figure 9

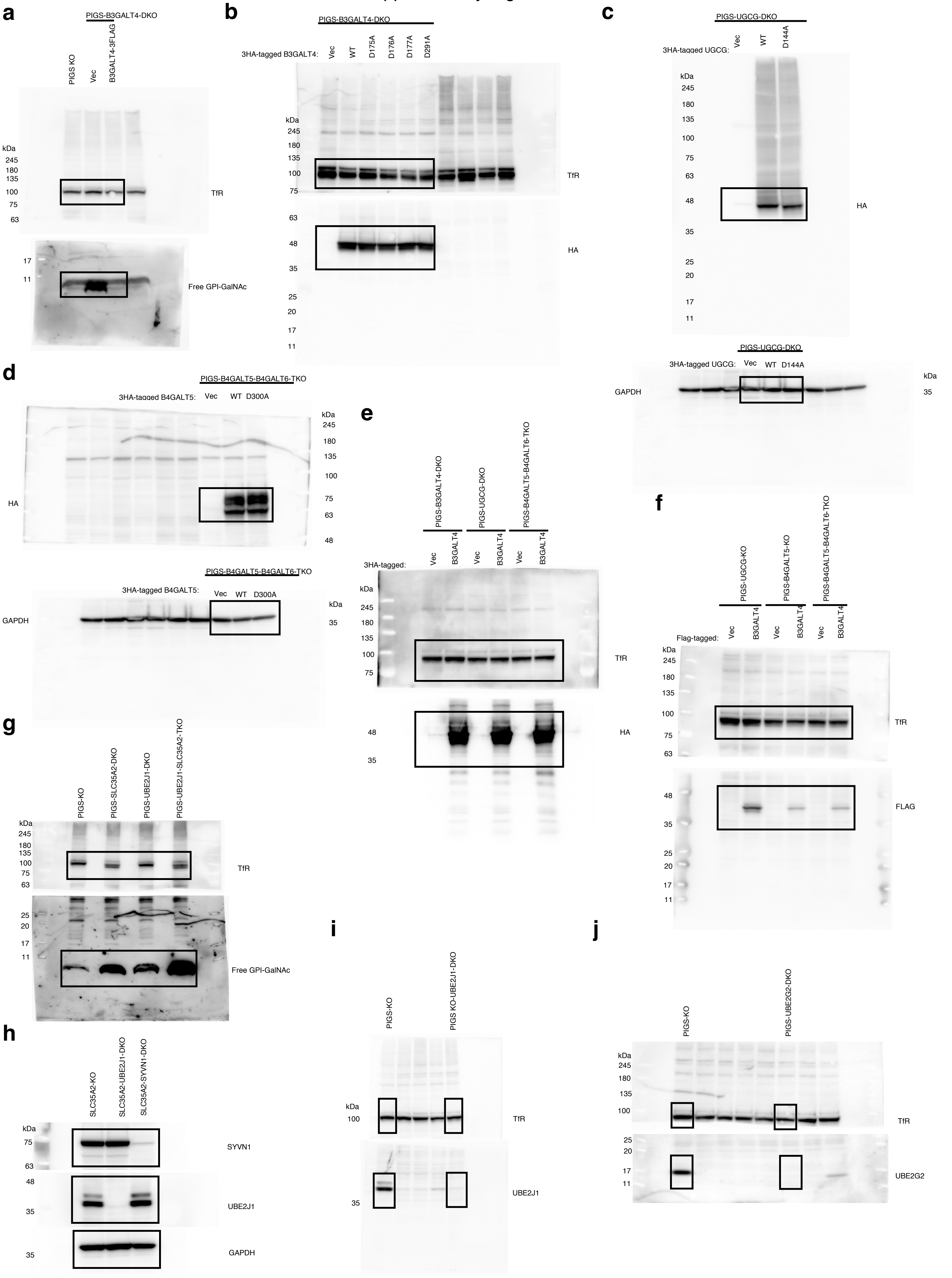

Supplementary Figure 9

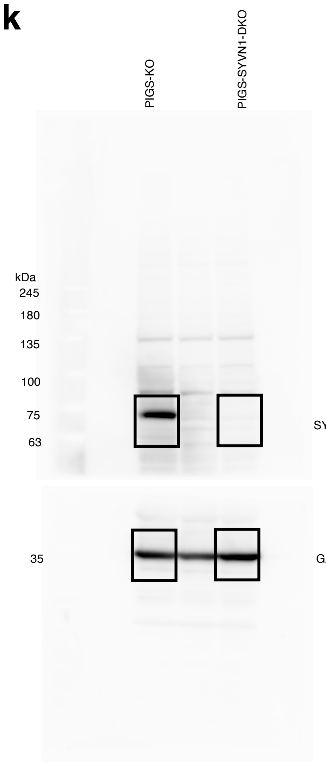

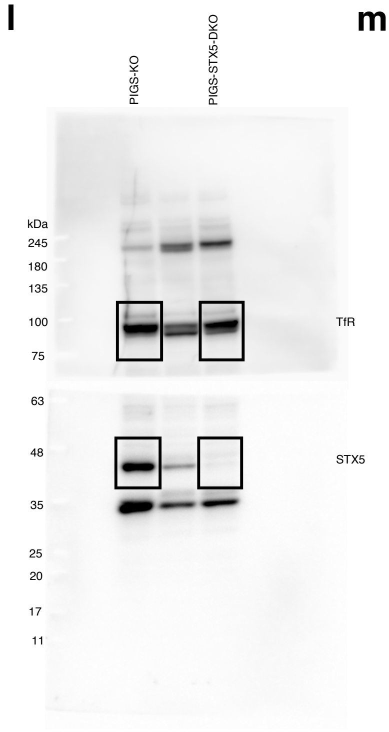

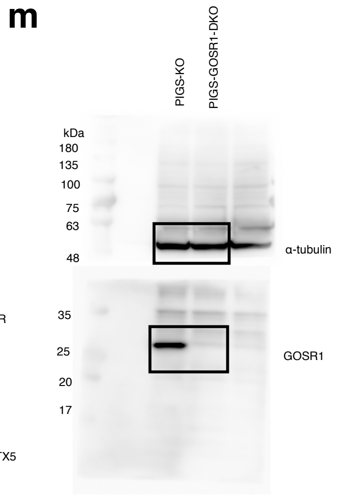

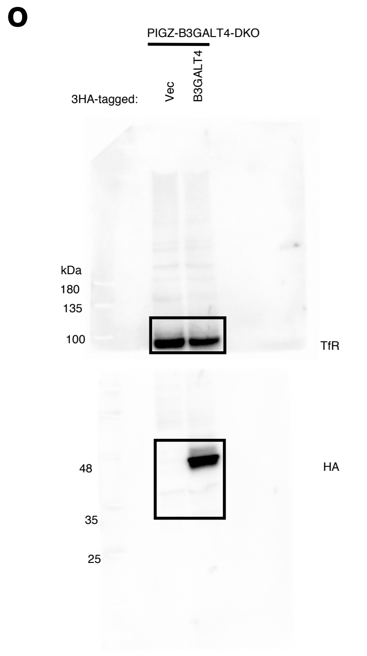

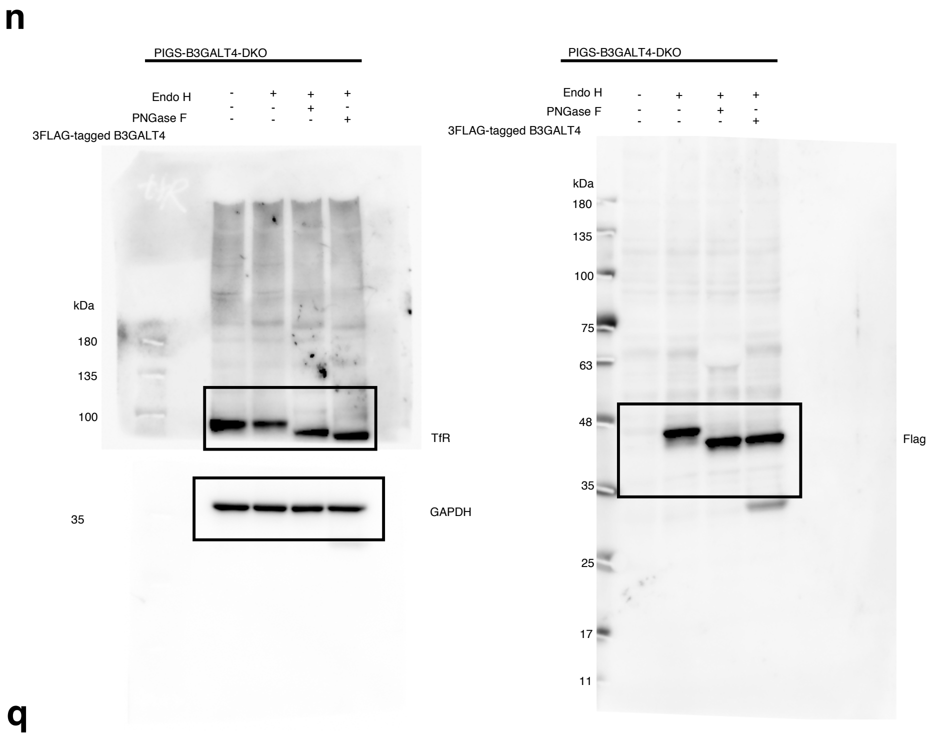

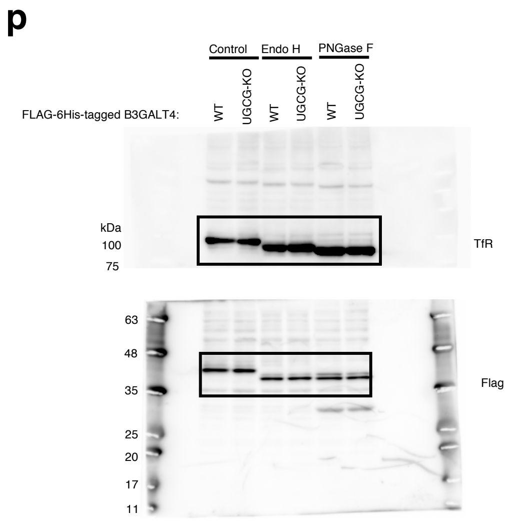

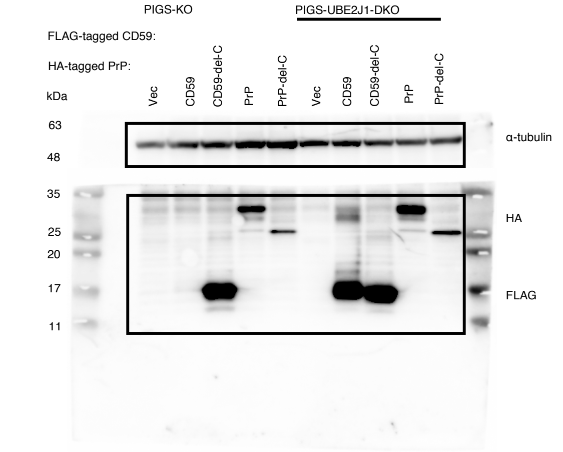

Supplementary Figure 9. Uncropped Western blots. **a** Related to Fig. 3d. **b** Related to Fig. 3g. **c** Related to Fig. 4b. **d** Related to Fig. 4e. **e** Related to Fig. 6a. **f** Related to Fig. 6d. **g** Related to Fig. 7c. **h** Related to Fig. 7f. **i** Related to Supplementary Fig. 1g. **j** Related to Supplementary Fig. 1h. **k** Related to Supplementary Fig. 1i. **l** Related to Supplementary Fig. 1j. **m** Related to Supplementary Fig. 1k. **n** Related to Supplementary Fig. 2a. **o** Related to Supplementary Fig. 2c. **p** Related to Supplementary Fig. 6f. **q** Related to Supplementary Fig. 8b.

Supplementary Tables

Supplementary Table 1: Genotyping of knockout cell lines. Related to Fig. 2, 3, and 4.

Cell Name	Gene	Sequence
HEK293-PIGS KO	B3GALT4	AAATTCCTGCTGACGTCCCACAGGCTGGACCCCTGG
HEK293-PIGS-B3GALT4 DKO	B3GALT4	AAATTCCTGCTG---15bp deletion---GACCCCTGG<br>AAATTCCTGCTGACGTCCCCACAGGCTGGACCCCTGG
HEK293-PIGS KO	UNC50	AAAGCTTCTCCTTTGGGTTGTACTCATAGATTGTGTAGGCGTTGGTCTTCTGATAGCAACTTTAATGTG
HEK293-PIGS-UNC50 DKO	UNC50	AAAGCTTCTCCTTT-GGTTGTACTCATAGATTGTGTAGGCGTTGGTCTTCTGATAGCAACTTTAATGTG<br>AAAGCTTCTCCTTTGG--4bp deletion--TACTCATAGATTGTGTAGGCGTTGGTCTTCTGATAGCAACTTTAATGTG
HEK293+HFGF-CD59	PIGZ	AGTGCTGTGGTGTCTCCTTCCGCAGACGGGCTATGTGCACCCAGATGAGTTCTTCCAGT
HEK293+HFGF-CD59	B3GALT4	ATGGGAAATTCCTGCTGACGTCCCACAGGCTGGACCCCTGGAAGATGCAGGA
HEK293-PIGZ-B3GALT4 DKO+HFGF-CD59	PIGZ	AGTGCTGTGGTGTCTCCTTCCG-AGACGGGCTATGTGCACCCAGATGAGTTCTTCCAGT<br>AGTGCTGTGGTGTCTCCTTCCGCCAGACGGGCTATGTGCACCCAGATGAGTTCTTCCAGT
HEK293-PIGZ-B3GALT4 DKO+HFGF-CD59	B3GALT4	ATGGGAAATTCCTGCTGACGTCCCCACAGGCTGGACCCCTGGAAGATGCAGGA
HEK293-PIGS KO	B4GALT5	GTCCCGGATCAGAATGCCTTGGGCTT
HEK293-PIGS-B4GALT5 DKO	B4GALT5	GTCCCGGATCAGAATTTGGCTTTTGTCTCGAGAAGATATGAGTGCAAAAAATTGGTGCCCGTTGGTTGGGCTT<br>GTCCCGGATCAGAACGCTTGGCGCACAATATAGAGGGGGAGGTTTTCAGCCATGGAGACCGTTTTTTCGGAGCGCAAGGCGCCCCCCCGTGGGCTT
HEK293-PIGS-B4GALT5 DKO	B4GALT6	TCAAACAACAACGTATCTCCCGG
HEK293-PIGS-B4GALT5-B4GALT6 TKO	B4GALT6	TCAAACAACAACGTATCTCCCCGG
HEK293-PIGS KO	A4GALT	AGCGGGTCTGCACCCTGTTCATCATCGGCTTCAAGTTCACGTTTTTCGTCTCCATCATGATCTACTGGCACGTTGTGGGAGAGCCCAAGGAGAAAGGGCAGCTCTATAACCTGCCAGCAGAGATCCCCTGCCCCACCTTGACACCCCCCACCCCACCCTCCCACGGCCCCACTCCAGGCAACATCTTCTTCCTGGAGACTTCAGACCGGACCAACCCCAACTTCCTGTTCATGTGCTCGGTGGAGTCGGCCGCCAGAACTCACCCCGAATCCCACGTGCTGGTCCTGATGAAAGGGCTTCCGGGTGGCAACGCCTCTCTG
HEK293-PIGS-A4GALT DKO	A4GALT	AGCGGGTCTGCACCCTGTTCATTCATCGG<br>AGCGGGTCTGCACCCTGTTCA---276bp deletion---GACCTCTGTTCAACGCCTCTCTG

DNA sequence changes are shown in red. PAM sites are underlined.

Supplementary Table 2: ESI-MS/MS analysis of GPI in CD59 derived from B3GALT4 knockout cells. Related to Fig. 3e, f and Supplementary Fig. 3.

Fragment	MW	Charge	m/z	%					
				WT	B3GALT4 KO	PIGZ-B3GALT4 DKO (1st experiment)	PIGZ-B3GALT4 DKO (2nd experiment)	PIGZ-B3GALT4 DKO + Vec	PIGZ-B3GALT4 DKO + B3GALT4
DLCNFNEQLE N-GPI	2529.90	3+	844.27	0	0	0.10	0	1.75	1.0018
		2+	1265.95	0	0	0	0	0.23	0
KDLCNFNEQ LEN-GPI	2657.93	3+	886.98	0	0	0	0.054	0	0
		2+	1329.97	0	0	0	0	0	0
DLCNFNEQLE N-GPI + Man4	2691.88	3+	898.29	0.046	0.0077	0	0	0	1.27
		2+	1346.94	0	0	0	0	0	0
KDLCNFNEQ LEN-GPI + Man4	2819.98	3+	940.99[A]	0.73	0.92	0	1.01	0	0
		2+	1410.99	0	0	0	0	0	0
DLCNFNEQLE N-GPI + GalNAc	2732.90	3+	911.98[B]	49.75	56.55	52.16	57.09	49.93	0.89
		2+	1367.47	4.75	4.39	6.26	11.92	3.84	0
KDLCNFNEQ LEN-GPI + GalNAc	2861.01	3+	954.68	15.72	19.58	41.48	29.67	24.22	0.80
		2+	1431.50	0.18	0.57	0	0.26	1.68	0
DLCNFNEQLE N-GPI + GalNAc + Hex*	2894.96	3+	965.99[D]	20.78	13.95	0	0	18.35[G]	36.54[H]
		2+	1448.48	0.77	0.023	0	0	0	0
KDLCNFNEQ LEN-GPI + GalNAc + Hex*	3023.06	3+	1008.69	4.75	4.01	0	0	0	28.87
		4+	756.76	0	0	0	0	0	0.72
DLCNFNEQLE N-GPI + GalNAc + Hex + Sia	3186.06	3+	1063.02[C]	1.14	0	0	0	0	3.85
		4+	797.51	0	0	0	0	0	0
KDLCNFNEQ LEN-GPI + GalNAc + Hex + Sia	3314.15	3+	1105.72	0.20	0	0	0	0	9.73
		4+	829.54	0	0	0	0	0	0.60
DLCNFNEQLE N-GPI + GalNAc + Gal + Man4	3057.02	3+	1020.01[E]	1.18	0	0	0	0	2.91
		4+	765.25	0.024	0	0	0	0	0
KDLCNFNEQ LEN-GPI + GalNAc + Gal + Man4	3185.11	3+	1062.70	0	0	0	0	0	5.59
		4+	797.28	0	0	0	0	0	0.83
DLCNFNEQLE N-GPI + GalNAc + Gal + Sia + Man4	3348.11	3+	1117.04[F]	0	0	0	0	0	6.39
		4+	838.03	0	0	0	0	0	0
KDLCNFNEQLE N-GPI + GalNAc + Gal + Sia + Man4	3476.21	3+	1159.74	0	0	0	0	0	0
		4+	870.05	0	0	0	0	0	0

GPI-containing C-terminal peptides of CD59 derived from PI-PLC-treated WT (HEK293) cells, B3GALT4-KO cells, and B3GALT4-PIGZ DKO cells transfected with vector only or with B3GALT4 cDNA. *Man4 or Gal. [A], [B], [C], [E], and [F] shown in Supplementary Fig. 3a, b, c, g, and h, respectively. [D] shown in Supplementary Fig. 3e and f. [G] is DLCNFNEQLEN-GPI + GalNAc + Man4. [H] is DLCNFNEQLEN-GPI + GalNAc + Gal.

Supplementary References

1. Kumar, S., Stecher, G. & Tamura, K. MEGA7: molecular evolutionary genetics analysis version 7.0 for bigger datasets. *Mol. Biol. Evol.* **33**, 1870–1874 (2016).
2. Waterhouse, A. M., Procter, J. B., Martin, D. M. A., Clamp, M. & Barton, G. J. Jalview Version 2—a multiple sequence alignment editor and analysis workbench. *Bioinformatics* **25**, 1189–1191 (2009).
3. Uhlén, M. *et al.* Tissue-based map of the human proteome. *Science (80-.).* **347**, 1260419 (2015).
