# Supplementary material for: Cross-talks of glycosylphosphatidylinositol biosynthesis with glycosphingolipid biosynthesis and ER-associated degradation
Source: Nat Commun. 2020 Feb 13;11:860. doi: 10.1038/s41467-020-14678-2 (PMC7018848; doi:10.1038/s41467-020-14678-2)
Supplement: Supplementary file 3 — Description of Additional Supplementary Files [file 41467_2020_14678_MOESM3_ESM.docx]

**Description of Supplementary Files**

**File Name: Supplementary Data 1**

**Description:** Oligonucleotides used in this study. Sequences of guide RNAs, primers for cloning, site-directed mutagenesis, genotyping, and qRT-PCR are listed.

**File Name: Supplementary Data 2**

**Description:** Guide RNA counts from CRISPR screen. Guide RNA counts from the unsorted cells (control) and the sorted cells (sort3) are listed. Related to Fig. 1.

**File Name: Supplementary Data 3**

**Description:** Gene scores in unsorted versus sort3 cells. Genes are listed based on gene scores. Top-ranking genes are categorized by functions in colors. Related to Fig. 1.

**File Name: Supplementary Data 4**

**Description:** Transcriptome Profiling with Microarrays. Expression levels and fold changes of 36 genes of GPI pathway in PIGS-KO and PIGS-UBE2J1-DKO HEK293 cells are shown. Related to Fig. 7h.
