# Supplementary material for: Cross-talks of glycosylphosphatidylinositol biosynthesis with glycosphingolipid biosynthesis and ER-associated degradation
Source: Nat Commun. 2020 Feb 13;11:860. doi: 10.1038/s41467-020-14678-2 (PMC7018848; doi:10.1038/s41467-020-14678-2)
Supplement: Supplementary file 8 — Reporting Summary [file 41467_2020_14678_MOESM8_ESM.pdf]

nature research

Corresponding author(s): Taroh Kinoshita

Last updated by author(s): Jan 25, 2020

Reporting Summary

Nature Research wishes to improve the reproducibility of the work that we publish. This form provides structure for consistency and transparency in reporting. For further information on Nature Research policies, see Authors & Referees and the Editorial Policy Checklist .

Statistics

For all statistical analyses, confirm that the following items are present in the figure legend, table legend, main text, or Methods section.

n/a	Confirmed	
☐	☒	The exact sample size (*n*) for each experimental group/condition, given as a discrete number and unit of measurement
☐	☒	A statement on whether measurements were taken from distinct samples or whether the same sample was measured repeatedly
☐	☒	The statistical test(s) used AND whether they are one- or two-sided<br>*Only common tests should be described solely by name; describe more complex techniques in the Methods section.*
☒	☐	A description of all covariates tested
☐	☒	A description of any assumptions or corrections, such as tests of normality and adjustment for multiple comparisons
☐	☒	A full description of the statistical parameters including central tendency (e.g. means) or other basic estimates (e.g. regression coefficient) AND variation (e.g. standard deviation) or associated estimates of uncertainty (e.g. confidence intervals)
☐	☒	For null hypothesis testing, the test statistic (e.g. *F*, *t*, *r*) with confidence intervals, effect sizes, degrees of freedom and *P* value noted<br>*Give P values as exact values whenever suitable.*
☒	☐	For Bayesian analysis, information on the choice of priors and Markov chain Monte Carlo settings
☒	☐	For hierarchical and complex designs, identification of the appropriate level for tests and full reporting of outcomes
☐	☒	Estimates of effect sizes (e.g. Cohen's *d*, Pearson's *r*), indicating how they were calculated

Our web collection on statistics for biologists contains articles on many of the points above.

Software and code

Policy information about availability of computer code

Data collection	BD FACSDiva was used to collect flow cytometry data; ZEN 3.0 was used to collect 3D-SIM images; Olympus Fluoview FV10-ASW 3.1 was used to collect confocal images; and Thermo X calibur 2.1.0.1140 was used to collect LC-ESI-MS/MS data.
Data analysis	GraphPad Prism 7 was used for statistical analysis and graph output; ImageJ and Olympus FV10-ASW 4.2 Viewer were used for confocal image analysis; FlowJo 10 was used for FACS data analysis; TBtools (v0.665) was used to generate Heat map; MAGeCK v0.5.6 was used for identification of CRISPR-screen hits; Open-Source PyMOL 2.1 was used for figure output of the predicted protein structure; Thermo Xcalibur 2.2 was used for LC-ESI-MS/MS data analysis; JustTLC was used for quantitative analysis of HPTLC data, and Affymetrix Transcriptome Analysis Console 4.1 was used for analyzing microarray data.

For manuscripts utilizing custom algorithms or software that are central to the research but not yet described in published literature, software must be made available to editors/reviewers. We strongly encourage code deposition in a community repository (e.g. GitHub). See the Nature Research guidelines for submitting code & software for further information.

Data

Policy information about availability of data

All manuscripts must include a data availability statement. This statement should provide the following information, where applicable:

- Accession codes, unique identifiers, or web links for publicly available datasets
- A list of figures that have associated raw data
- A description of any restrictions on data availability

The source data underlying Figs 2, 3b and h, 4c-d and f-i, 5b, 6c and e, 7a-b and d-e, and Supplementary Figs 5b and g-h, 6a-b, and 8c-d are provided as a Source Data file. Uncropped western blot images are available in Supplementary Fig. 9. Sequencing data are available in Supplementary Data 2. The microarray data are available in Supplementary Data 4 and from the GEO database under accession number GSE140855 [https://www.ncbi.nlm.nih.gov/geo/query/acc.cgi?acc=GSE140855] (Microarray of HEK293-PIGS-KO and HEK293-PIGS-UBE2J1-DKO cells). The mass spectrometry proteomics data have been deposited to the

ProteomeXchange Consortium via the PRIDE76 partner repository with the dataset identifier PXD014226 [http://proteomecentral.proteomexchange.org/cgi/GetDataset?ID=PXD014226] (project name Determination of GPI structure of human CD59 in HEK293 cells). All other data that support the findings of this study are available from the corresponding author upon reasonable request.

Field-specific reporting

Please select the one below that is the best fit for your research. If you are not sure, read the appropriate sections before making your selection.

- [x] Life sciences
- [ ] Behavioural & social sciences
- [ ] Ecological, evolutionary & environmental sciences

For a reference copy of the document with all sections, see nature.com/documents/nr-reporting-summary-flat.pdf

Life sciences study design

All studies must disclose on these points even when the disclosure is negative.

Sample size	No statistical method was used to predetermine the sample size. Typically, for knockout glycosyltransferase genes or validation of genes by knockout, multiple (2-5) HEK293 or CHO cell clones were obtained and confirmed for knockout by FACS analysis, Western blotting or Sanger sequencing. One clone from the most representative clones were chosen for rescue experiments.
Data exclusions	No data were excluded from the analysis.
Replication	All FACS analysis were repeated independently. All attempts at replication were successful.
Randomization	This paper does not include comparison studies among groups, so this issue does not apply.
Blinding	Investigators were not blinded to group allocation

Reporting for specific materials, systems and methods

We require information from authors about some types of materials, experimental systems and methods used in many studies. Here, indicate whether each material, system or method listed is relevant to your study. If you are not sure if a list item applies to your research, read the appropriate section before selecting a response.

Materials & experimental systems

n/a	Involved in the study	
[ ]	[x]	Antibodies
[ ]	[x]	Eukaryotic cell lines
[x]	[ ]	Palaeontology
[x]	[ ]	Animals and other organisms
[x]	[ ]	Human research participants
[x]	[ ]	Clinical data

Methods

n/a	Involved in the study	
[x]	[ ]	ChIP-seq
[ ]	[x]	Flow cytometry
[x]	[ ]	MRI-based neuroimaging

Antibodies

Antibodies used	The following antibodies were used in this study:<br>Mouse monoclonal anti-Toxoplasma gondii GPI anchor (clone T5 4E10) antibody (T5 mAb) was a gift from Dr. Jean François Dubremetz (Montpellier University, France). T5 mAb (# NR-50267) is now available from BEI Resources, NIAID, NIH.<br>CD59 (clone 5H8) was a gift from Dr. M. Tomita and Dr. Y. Sugita.<br>DYKDDDDK (FLAG) (# 014-22383, clone 1E6, Wako), TfR (# 13-6800, clone H68.4, Thermo) , GAPDH (# MA1-22670, clone GA1R, Thermo), Lactosylceramide (CD17) (# MA1-10118, clone MEM-68, Thermo), GM130 (# ab76154, clone EP892Y, Abcam), UBE2J1 (# sc-377002, clone B-6, Santa Cruz), UBE2G2 (# sc-393780, clone D-4, Santa Cruz), GOSR1 (# sc-271551, clone F11, Santa Cruz), Syntaxin 5 (# sc-365124, clone B8, Santa Cruz), SYVN1 (# 13473-1-AP, Proteintech), B4GALT1 (# HPA010807, Atlas), TGN46 (# AHP500G, Bio-Rad), GM130 (# 610822, clone 35, BD), HA (# 3724, clone C29F4, CST), HA (# H3663, clone HA-7, Sigma), α-Tubulin (# T9026, clone clone DM1A, Sigma), and FLAG (FITC-conjugated) (# F4049, clone M2, Sigma).
Validation	All antibodies used in our study have been validated. Detailed information could be found on the manufactures' website as listed below. T5 mAb has been validated by knockout of GPI pathway genes, and Lactosylceramide (CD17) has been validated by knockout of ST3GAL5. Antibodies including DYKDDDDK (FLAG, Wako), CD59, HA, and FLAG (Sigma) used in our study have been validated by overexpress. Antibodies including UBE2J1, UBE2G2, GOSR1, Syntaxin 5, and SYVN1 have been validated by our experiments in this manuscript using knockout by CRISPR/Cas9 method.<br>DYKDDDDK (FLAG), https://labchem-wako.fujifilm.com/europe/product/detail/W01W0101-2238.html<br>TfR, https://www.thermofisher.com/cn/zh/antibody/product/Transferrin-Receptor-Antibody-clone-H68-4-Monoclonal/13-6800<br>GAPDH, https://www.thermofisher.com/cn/zh/antibody/product/GAPDH-Loading-Control-Antibody-clone-GA1R-Monoclonal/

MA5-15738
Lactosylceramide (CD17), https://www.thermofisher.com/cn/zh/antibody/product/CD17-Antibody-clone-MEM-68-Monoclonal/MA1-10118
GM130 (Abcam), https://www.abcam.com/gm130-antibody-ep892y-cis-golgi-marker-ab52649.html
UBE2J1, https://www.scbt.com/p/ube2j1-antibody-b-6?productCanUrl=ube2j1-antibody-b-6&_requestid=1151038
UBE2G2, https://www.scbt.com/p/ube2g2-antibody-d-4?productCanUrl=ube2g2-antibody-d-4&_requestid=1150922
GOSR1, https://www.scbt.com/p/gs28-antibody-f-11?productCanUrl=gs28-antibody-f-11&_requestid=1150270
Syntaxin5, https://www.scbt.com/p/syntaxin-5-antibody-b-8?productCanUrl=syntaxin-5-antibody-b-8&_requestid=302064
SYVN1, https://www.ptglab.com/Products/SYVN1-Antibody-13473-1-AP.htm
B4GALT1, https://www.atlasantibodies.com/products/antibodies/primary-antibodies/triple-a-polyclonals/b4galt1-antibody-hpa010807/
TGN46, https://www.bio-rad-antibodies.com/polyclonal/human-tgn46-antibody-ahp500.html?f=purified
GM130 (BD), https://www.bdbiosciences.com/us/reagents/research/antibodies-buffers/cell-biology-reagents/cell-biology-antibodies/purified-mouse-anti-gm130-35gm130/p/610822
HA (CST), https://www.cellsignal.com/products/primary-antibodies/ha-tag-c29f4-rabbit-mab/3724
HA (Sigma), https://www.sigmaaldrich.com/catalog/product/sigma/h3663?lang=en&region=US
α-Tubulin, https://www.sigmaaldrich.com/catalog/product/sigma/t9026?lang=en&region=US
FLAG (FITC-conjugated), https://www.sigmaaldrich.com/catalog/product/sigma/f4049?lang=en&region=US

Eukaryotic cell lines

Policy information about cell lines

Cell line source(s)	HEK293 cells (ATCC CRL-1573), HeLa cells (ATCC CCl-2), and CHO K1 cells (ATCC CCL-61) were used for this study.
Authentication	None of the cell lines were authenticated.
Mycoplasma contamination	The cell lines are not tested for Mycoplasma contamination.
Commonly misidentified lines (See ICLAC register)	None of the cell lines used are listed in the ICLAC database.

Flow Cytometry

Plots

Confirm that:

- [x] The axis labels state the marker and fluorochrome used (e.g. CD4-FITC).
- [x] The axis scales are clearly visible. Include numbers along axes only for bottom left plot of group (a 'group' is an analysis of identical markers).
- [x] All plots are contour plots with outliers or pseudocolor plots.
- [x] A numerical value for number of cells or percentage (with statistics) is provided.

Methodology

Sample preparation	Cells stained with T5 mAb, anti-CD59 and anti-CD17 (LacCer) in FACS buffer (PBS containing 1% BSA and 0.1% NaN3) were incubated on ice for 25 min. Cells were then washed twice in FACS buffer followed by staining with Alexa Fluor 488 or 647-conjugated goat anti-mouse IgM for T5 mAb and anti-CD17, and APC or PE-conjugated streptavidin for biotin-labeled anti-CD59 in FACS buffer. To analyze the glycosylation profiles of cell surfaces, cells were stained by Alexa Fluor 488-conjugated CTxB or Alexa Fluor 647-conjugated lectins in FACS buffer containing 1 mM CaCl2, 1 mM MnCl2, and 1 mM MgCl2 on ice for 15 min. After twice washing by FACS buffer, samples were analyzed within 1 hour.
Instrument	BD FACSCanto II
Software	BD FACSDiva was used to collect data; and FlowJo was used to analyze the data.
Cell population abundance	We only used cultured cell lines, so cell population abundance was always 100%. At least 30000 cells were analyzed for each sample.
Gating strategy	We used cell lines and gating of different populations does not apply to this study.

- [ ] Tick this box to confirm that a figure exemplifying the gating strategy is provided in the Supplementary Information.
